# Haemothorax and Thoracic Spine Fractures in the Elderly

**DOI:** 10.1155/2012/162064

**Published:** 2012-09-02

**Authors:** Michael A. Masteller, Aakash Chauhan, Harsha Musunuru, Mark M. Walsh, Bryan Boyer, Joseph A. Prahlow

**Affiliations:** ^1^Indiana University School of Medicine, South Bend Campus, South Bend, IN 46556, USA; ^2^Department of Orthopaedic Surgery, Allegheny General Hospital, Pittsburgh, PA 15212, USA; ^3^Department of Emergency Medicine, Memorial Hospital of South Bend, South Bend, IN 46601, USA; ^4^Memorial Hospital of South Bend, South Bend, IN 46601, USA; ^5^South Bend Medical Foundation, South Bend, IN 46601, USA

## Abstract

Both osteoporotic fractures and pleural effusions are frequently observed in medicine. However, rarely does one associate a hemorrhagic pleural effusion with a thoracic spinal fracture when the patient has not sustained massive trauma. In this paper, we discuss two cases where seemingly insignificant low-energy trauma precipitated massive haemothoraces in elderly patients with underlying osteoporosis, ultimately resulting in their immediate causes of death. This paper serves to remind health care professionals of the importance of using caution when moving elderly patients as well as to consider thoracic spinal fracture as a potential explanation for a hemorrhagic pleural effusion of undetermined etiology.

## 1. Introduction

Osteoporosis is a common disease in the elderly, currently affecting more than 10 million people in the United States [1]. The decrease in bone density compromises its structural integrity, resulting in an increased rate of fractures. The most common locations for these types of fractures are the spine, humerus, wrist, pelvis, and hip [2]. Many of these fractures are insufficiency fractures, a subgroup of stress fractures where a break is due to normal or physiologic stress upon weakened bone.

We present two cases of minimal low-energy trauma associated with thoracic spine fractures as the cause of haemothoraces in geriatric patients. While a number of haemothoraces from thoracic spinal fractures related to massive trauma have been reported [3–5], a review of the literature for fractures from low-energy traumatic events failed to reveal a single case. The purpose of this case study is to report on the potential risk of fatal haemothorax due to low-energy traumatic spinal insufficiency fractures.

## 2. Case Report

### 2.1. ****1st Patient Case

**Figure 1:**
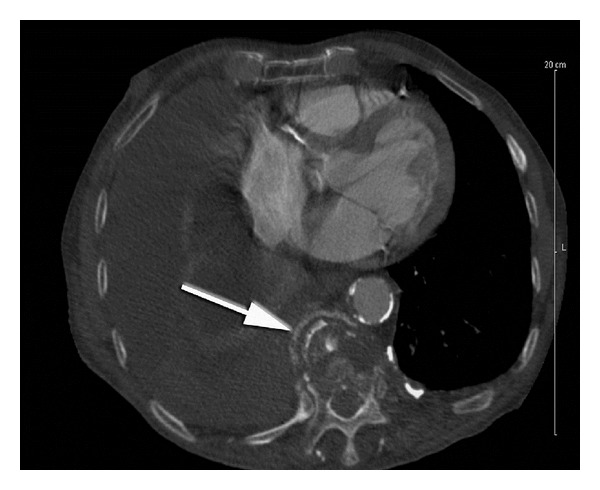
CT of case report 1 showing location of thoracic fracture.

A 93-year-old white male presented to the emergency department (ED) with a chief complaint of weakness and recurrent falls combined with a history of osteoporosis, dementia, and an inability to care for himself. Upon examination, decreased breath sounds in the right lung were noted, and a chest X-ray revealed a large right pleural effusion. A computed tomography (CT) scan of the chest confirmed the suspicion of a pleural effusion and revealed multiple rib fractures at various stages of healing, a clavicular fracture, L1-L2 vertebral body compression fractures, and a large lucency at the anterior aspect of the approximate T10 vertebral body (Figure 1). The patient underwent a thoracentesis yielding 1000 mL of hemorrhagic fluid.

**Figure 2:**
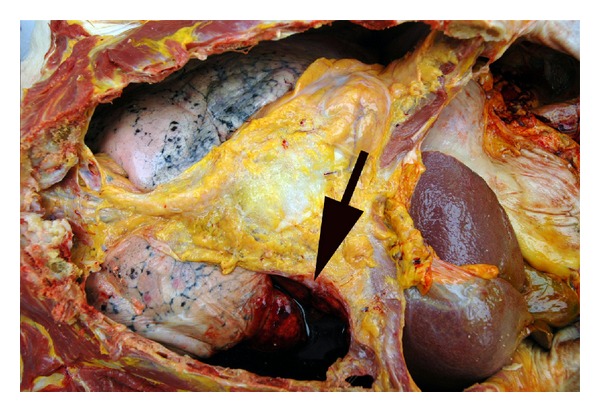
A right-sided haemothorax visualized at autopsy, following initial incision and chest plate removal.

**Figure 3:**
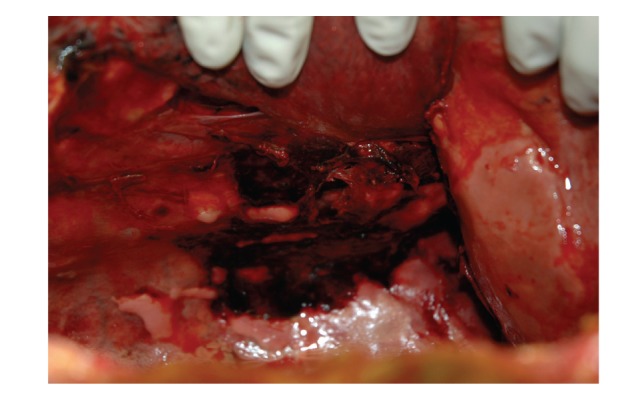
A closer view of the site of hemorrhage along the right lateral edge of the lower thoracic spinal column.

**Figure 4:**
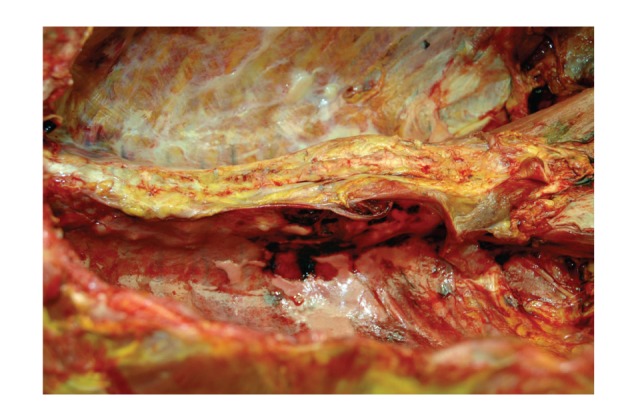
A view of the thoracic spinal column in situ, after organ viscera removal (including aorta), demonstrating hemorrhage associated with the lower thoracic spinal column fracture.

**Figure 5:**
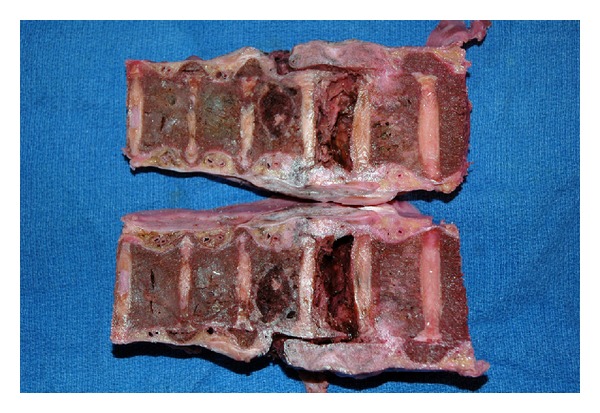
A section of the lower thoracic spinal column, after removal, formalin fixation, and longitudinal sectioning, demonstrating fractures of the 10th and 11th vertebral bodies.

Due to the patient's poor nutritional status and the family's decision to decline further treatment, palliative care was initiated. The patient expired four days after admission. The patient had a history of asbestos exposure in the distant past, and, as a result, the family requested an autopsy to determine whether mesothelioma was the cause of the hemorrhagic pleural effusion.Autopsy showed the body of a well-developed, emaciated adult white male weighing 110 lb and 67.5 inches tall (BMI = 17.0 kg/m^2^). Examination of the thoracic cavity revealed recent fractures of his left 2nd and 3rd ribs as well as multiple healing fractures bilaterally. No blood was emanating from any of these fractures. Most notably, however, was a 2000 mL haemothorax (Figure 2) which had arisen from fractures of the 10th and 11th thoracic vertebrae with associated laceration of the posteromedial aspect of the right parietal pleural lining, overlying the fracture site (Figures 3, 4 and 5). Adherent blood clots were present near the laceration site as well as on the lung subjacent to the fracture site. The overlying large-caliber vasculature (aorta, vena cava, and branches) was intact and uninjured. Although fibrous pleural plaques consistent with asbestos exposure were observed, no evidence of mesothelioma was present, either grossly or by microscopic examination.

Given the autopsy findings, as well as his history of recurrent falls and underlying osteoporosis, his thoracic spine fractures were most likely sustained from a recent fall and ultimately determined to be the immediate cause of death related to a massive haemothorax. The manner of death was ruled an accident.

### 2.2. ****2nd Patient Case

**Figure 6:**
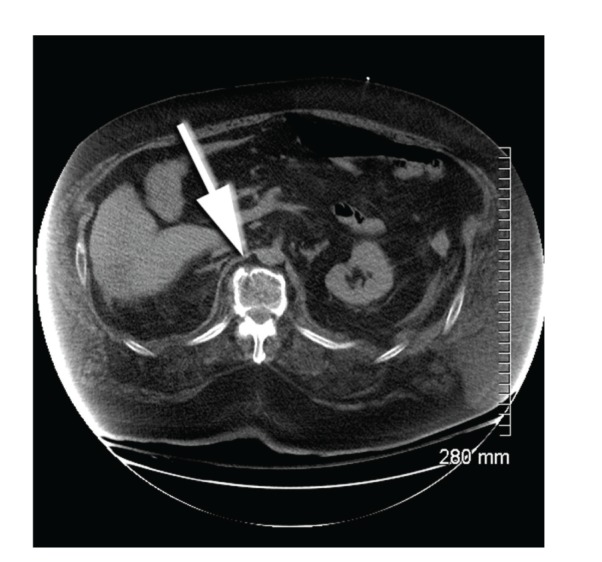
CT of case report 2 showing location of thoracic fracture.

A 71-year-old white male status post sigmoid colectomy for removal of a colorectal carcinoma developed acute respiratory distress postoperatively, after being moved from the operating table, and was reintubated. Attempts to wean the patient from the ventilator resulted in continued distress and subsequent respiratory failure. The patient quickly declined and progressed to cardiopulmonary arrest. After approximately an hour of resuscitation efforts and stabilizing the patient, he was transferred to the intensive care unit (ICU), where a chest X-ray revealed a large right pleural effusion of unknown origin. A chest tube was placed and 3000 mL of hemorrhagic pleural fluid was withdrawn. A subsequent chest CT did not reveal an obvious source of hemorrhage (Figure 6). As time progressed and the patient remained nonresponsive after numerous interventions, it became exceedingly clear that the patient had suffered a severe irreversible anoxic encephalopathy from the prolonged cardiopulmonary arrest. The family agreed to withdraw life support secondary to the patient's irreversible medical condition.

**Figure 7:**
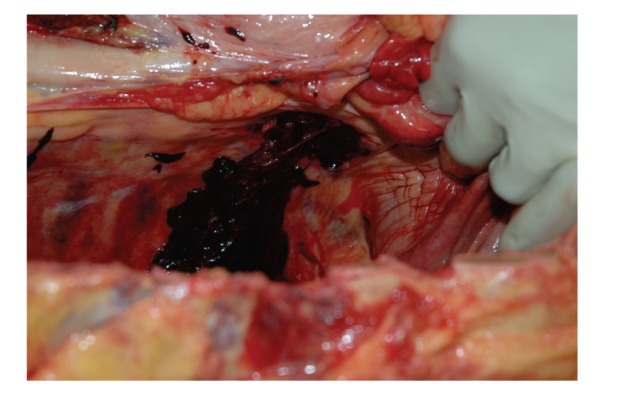
Site of localized hemorrhage arising from the lateral aspect of the lower thoracic spinal column, viewed from within the right pleural cavity with right hemidiaphragm being displaced downward by the gloved hand.

**Figure 8:**
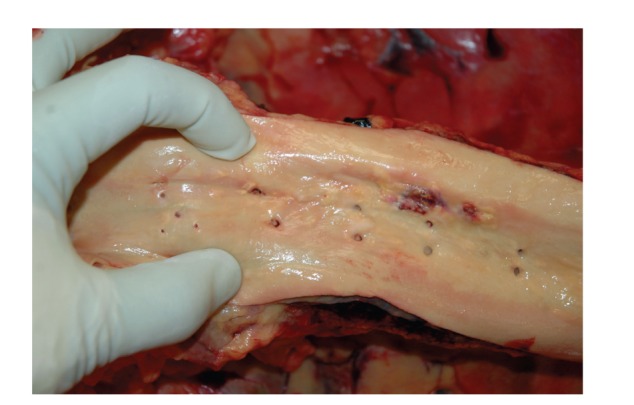
In situ examination of the aorta (opened anteriorly). Note the presence of a small area of severe atherosclerotic plaque. There was, however, no associated traumatic aortic injury.

**Figure 9:**
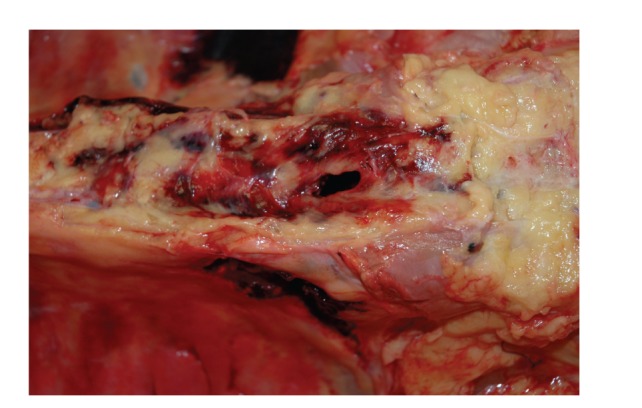
After removing the aorta, soft tissue hemorrhage is evident over the lower thoracic spine.

**Figure 10:**
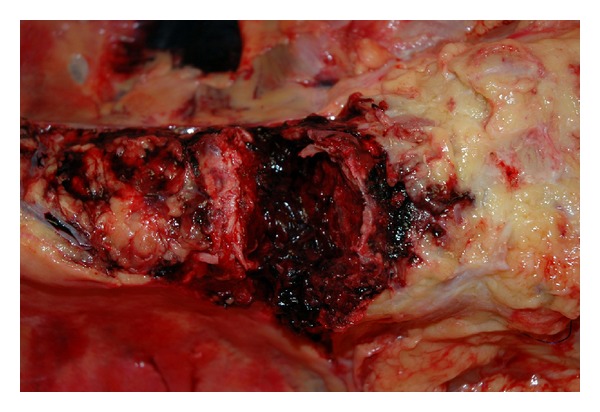
After dissecting away additional overlying soft tissue, a large fracture of the 11th thoracic vertebral body is clearly evident.

Autopsy showed the body of a well-developed, morbidly obese adult white male weighing 330 lb and 74 inches tall (BMI = 42.4 kg/m^2^). Examination of the thoracic cavity revealed 1000 mL of serosanguineous fluid with clotted blood within the right chest cavity as well as 400 mL of serosanguineous fluid within the left chest cavity. This fluid originated from the posterior chest wall near the lower thoracic spinal column (Figure 7). Originally, an aortic injury was suspected, but the aorta was free of acute trauma, demonstrating only focal complicated atherosclerotic plaque (Figure 8). The hemorrhage arose from a fracture of the 11th thoracic vertebral body (Figures 9 and 10), presumably having occurred while moving the patient from the operating table to the hospital bed for transport to the recovery room, as no other sources of trauma could be identified. Microscopic examination of the spine demonstrated an insufficiency fracture secondary to osteoporosis. No evidence of malignancy was present.

Based upon the gross and microscopic findings in the decedent's autopsy, massive haemothorax from a fractured thoracic vertebra with underlying osteoporosis was the cause of death. The manner of death was ruled an accident.

## 3. Discussion

Over 750,000 vertebral compression fractures (VCFs) occur each year in the United States [1]. Of these, 20% occur in people above 70 years of age [16]. These VCFs account for an estimated 27% of all osteoporotic fractures [16–18]. Risk factors for osteoporosis include long-term glucocorticoid therapy, low body weight, family history of hip fracture, cigarette smoking, excessive alcohol intake, history of previous fracture, dietary calcium or vitamin D deficiency, and advancing age [19]. Trauma associated with VCFs occurs when the weight of the upper body exceeds the bones' capacity to support the load within the vertebral body. The force causes the anterior portion of the vertebral body to crush, forming a wedge fracture. This leads to a kyphotic deformity as the spine bends forward from the resultant loss in the anterior height [20]. Hemorrhage in VCFs occurs mainly from the azygos and hemiazygos vein, intercostal artery, and external vertebral venous plexus [9]. A paravertebral hematoma forms due to bleeding from those vessels. If the parietal pleura is undamaged, spontaneous hemostasis will likely occur [9]. In these two patient cases, osteoporosis led to an insufficiency fracture of the thoracic spine and as a result created an unstable environment by which the patients developed haemothoraces and rapid clinical deterioration. 

Table 1 provides a recap of many of the extensive differential diagnoses for pleural effusion. When discussing the causes of a pleural effusion in the elderly, haemothorax from a thoracic spinal fracture is not a common cause and certainly low on a list of differential diagnoses. However, in order to treat these patients effectively, clinicians must always consider this etiology, especially in those with risk factors or predisposing conditions.

In conclusion, we hope that this paper will serve as a reminder to physicians of the potential common and uncommon causes of haemothoraces. Haemothoraces from thoracic spinal fractures can often be overlooked, especially when it appears that little to no trauma has occurred. With the increasing elderly population and the consequential increasing incidence of osteoporosis, we expect cases similar to these to become more prevalent. Although such cases cannot always be avoided, the cause can often be ascertained in a timely manner when the appropriate diagnoses are considered, potentially preventing fatalities.

## Figures and Tables

**Table 1:** Potential causes of pleural effusions.

Cirrhosis [6]	Congestive heart failure [6]	Malignancies [7]
Drug-induced [6, 8]	Bacterial/viral pneumonia [6]	Pulmonary embolism [6]
Sarcoidosis [6]	Lupus [9]	Rheumatoid arthritis [10]
Pancreatitis [6]	Fungal infection [6]	Asbestos exposure [11]
Hemothorax [3–5]	Trapped lung [12]	Tuberculosis [10]
Empyema [6]	Hypothyroidism [6]	Urinothorax [6]
Constrictive pericarditis [6]	Yellow nail syndrome [6]	Chylothorax [6]
Meigs' syndrome [13]	Dressler's syndrome [14]	Boerhaave syndrome [15]
